# *Aronia melanocarpa* Juice Induces a Redox-Sensitive p73-Related Caspase 3-Dependent Apoptosis in Human Leukemia Cells

**DOI:** 10.1371/journal.pone.0032526

**Published:** 2012-03-08

**Authors:** Tanveer Sharif, Mahmoud Alhosin, Cyril Auger, Carole Minker, Jong-Hun Kim, Nelly Etienne-Selloum, Pierre Bories, Hinrich Gronemeyer, Annelise Lobstein, Christian Bronner, Guy Fuhrmann, Valérie B. Schini-Kerth

**Affiliations:** 1 UMR 7213 CNRS, Laboratoire de Biophotonique et Pharmacologie, Faculté de Pharmacie, Université de Strasbourg, Illkirch, France; 2 UMR 7200 CNRS, Laboratoire d'Innovation Thérapeutique, Faculté de Pharmacie, Université de Strasbourg, Illkirch, France; 3 UMR 7104 CNRS/U 964 INSERM, Institut de Génétique et de Biologie Moléculaire et Cellulaire, Université de Strasbourg, Illkirch, France; The University of Kansas Medical Center, United States of America

## Abstract

Polyphenols are natural compounds widely present in fruits and vegetables, which have antimutagenic and anticancer properties. The aim of the present study was to determine the anticancer effect of a polyphenol-rich *Aronia melanocarpa* juice (AMJ) containing 7.15 g/L of polyphenols in the acute lymphoblastic leukemia Jurkat cell line, and, if so, to clarify the underlying mechanism and to identify the active polyphenols involved. AMJ inhibited cell proliferation, which was associated with cell cycle arrest in G_2_/M phase, and caused the induction of apoptosis. These effects were associated with an upregulation of the expression of tumor suppressor p73 and active caspase 3, and a downregulation of the expression of cyclin B1 and the epigenetic integrator UHRF1. AMJ significantly increased the formation of reactive oxygen species (ROS), decreased the mitochondrial membrane potential and caused the release of cytochrome c into the cytoplasm. Treatment with intracellular ROS scavengers prevented the AMJ-induced apoptosis and upregulation of the expression of p73 and active caspase 3. The fractionation of the AMJ and the use of identified isolated compounds indicated that the anticancer activity was associated predominantly with chlorogenic acids, some cyanidin glycosides, and derivatives of quercetin. AMJ treatment also induced apoptosis of different human lymphoblastic leukemia cells (HSB-2, Molt-4 and CCRF-CEM). In addition, AMJ exerted a strong pro-apoptotic effect in human primary lymphoblastic leukemia cells but not in human normal primary T-lymphocytes. Thus, the present findings indicate that AMJ exhibits strong anticancer activity through a redox-sensitive mechanism in the p53-deficient Jurkat cells and that this effect involves several types of polyphenols. They further suggest that AMJ has chemotherapeutic properties against acute lymphoblastic leukemia by selectively targeting lymphoblast-derived tumor cells.

## Introduction

Natural products derived from plants have received considerable attention as potential cancer chemopreventive and chemotherapeutic agents over few decades. On the basis of epidemiological and animal studies, it has been reported that diets rich in fruits and vegetables are associated with a reduced rate of cancer mortality. Dietary phytochemicals consist of a wide variety of biologically active compounds which are known to exert their anticancer activity on the three stages of carcinogenesis, which include initiation, promotion and progression [1]. Natural products rich in polyphenols, such as green tea and red wine, have been shown to have strong chemopreventive and chemotherapeutic properties in different types of cancer cells [2], [3], [4]. Moreover, the polyphenol-induced cytotoxic effect appears to target specifically cancer cells [5], [6].

*Aronia melanocarpa* (Michx.) Elliott (Rosaceae) also known as black chokeberry is a shrub native from North America, which is now cultivated extensively in Europe [7]. *Aronia melanocarpa* juice (AMJ) is one of the richest sources of natural polyphenols; one liter of juice can contain up to 7 g of polyphenols [8]. AMJ has been shown to have numerous health benefits, including cardioprotective, hepatoprotective and antidiabetic activities [7]. Several *in vitro* and *in vivo* studies indicate that *Aronia melanocarpa* extracts have also anti-proliferative effects against several colon cancer cells [9], [10], [11]. Indeed, AMJ inhibited the growth and triggered apoptosis of human colon cancer HT-29 cells but had little effect on non-tumorigenic colonic NCM460 cells [9]. Moreover, cell proliferation inhibition of human colorectal carcinoma cell line (Caco-2) is associated with G_2_/M cell cycle arrest and a sharp up-regulation of the tumor suppressor carcinoembryonic antigen-related cell adhesion molecule 1 (CEACAM1) [11].

Although polyphenols are known for their antioxidant properties, several recent studies indicate that polyphenolic compounds such as (−)-epigallocatechin-3-gallate, genistein, resveratrol, and hispolon promote apoptosis of cancer cells by inducing a pro-oxidant response [12], [13], [14], [15]. However, the nature and the source of reactive oxygen species (ROS) produced in response to polyphenols remain poorly studied. A role for mitochondria-derived superoxide anions has been suggested in resveratrol-induced apoptosis in HT-29 human colorectal carcinoma cells [13], and a reduced glutathione antioxidant system in hispolon-induced apoptosis in human gastric cancer cells [14].

Although epidemiological studies have shown a relationship between regular consumption of natural polyphenolic-rich products from plants and a reduced risk of leukemia [16], [17], very few studies have evaluated their chemotherapeutic potential in the management of leukemias [3]. Therefore, the aim of the present study was to determine whether AMJ inhibits proliferation of the human acute lymphoblastic leukemia Jurkat cell line, and if so, to identify the underlying molecular mechanism in particular the role of ROS. In order to better appreciate the clinical relevance, the pro-apoptotic effect of AMJ was also studied in normal human primary T-lymphocytes and human primary lymphoblastic leukemia cells.

## Results

### AMJ decreases proliferation and causes G_2_/M cell cycle arrest in acute lymphoblastic leukemia Jurkat cells

**Figure 1:**
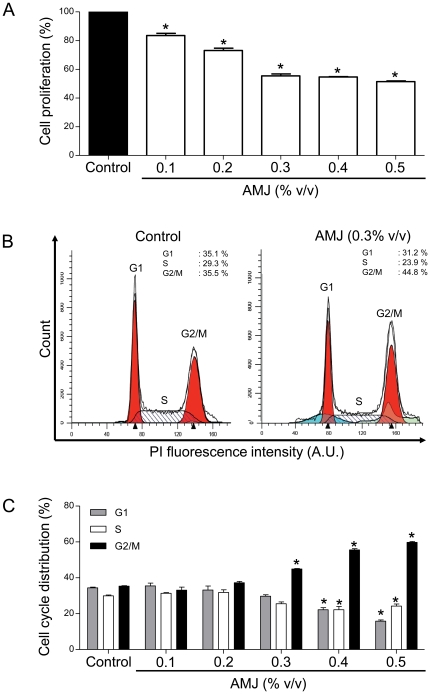
AMJ caused a concentration-dependent inhibition of cell proliferation and induction of G2/M cell cycle arrest in Jurkat cells. Cells were exposed to increasing concentrations of AMJ for 24 h. (A) cell proliferation rate was determined using MTS assay. The absolute value obtained for each AMJ-treated sample is expressed as percent relative to the absolute value obtained for the untreated sample and set at 100%. (B) and (C) cell cycle distribution was assessed by flow cytometry using the DNA fluorochrome PI detection assay. (B) shows representative DNA content histograms for treated (right panel) or untreated (left panel) cells. (C) Corresponding cumulative data. The number of cells in each mitosis phase is determined and expressed as percent relative to the total cell number. Values are shown as means ± S.E.M. (n = 3); *, *P<0.05* versus respective control.

As shown in Fig. 1A, AMJ decreased the percentage of living cells in a concentration-dependent manner with a significant effect (18% decrease) observed at 0.1% v/v. Since cell growth is associated with their ability to progress through the different phases of mitosis, the effect of AMJ exposition on the cell cycle phase distribution was examined. As shown in Fig. 1B and C, AMJ, at concentrations from 0.3% to 0.5% v/v, significantly increased accumulation of cells in the G_2_/M phase. Thus, AMJ inhibited the growth of acute lymphoblastic leukemia Jurkat cells by promoting their cell cycle arrest in G_2_/M phase.

### AMJ induces apoptosis and modulates the expression of proteins related to cell cycle and apoptosis in Jurkat cells

**Figure 2:**
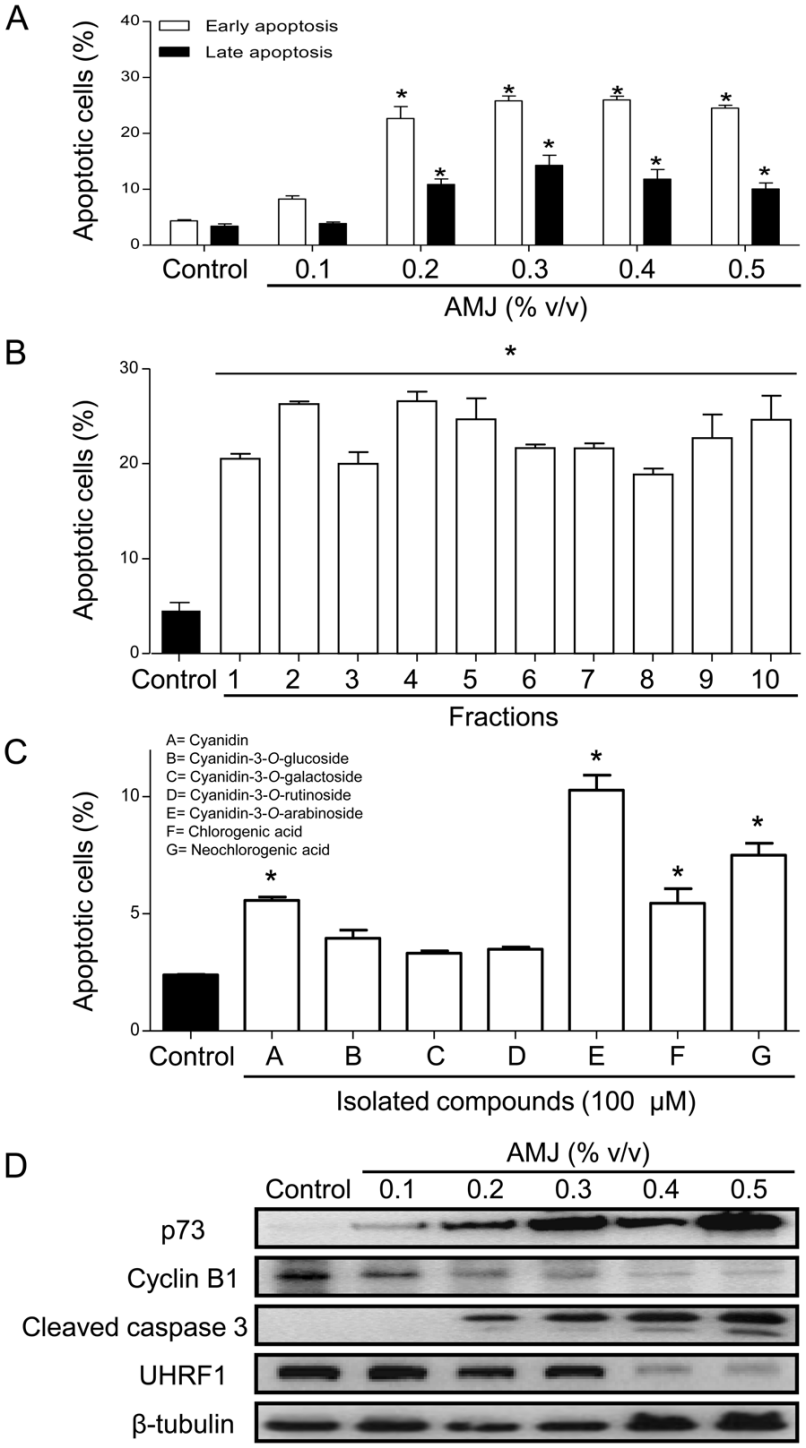
AMJ and AMJ-derived fractions induced a pro-apoptotic response in Jurkat cells. Cells were exposed either to increasing concentrations of AMJ, or AMJ-derived fractions (tested at 21.4 µg/mL, equivalent to the polyphenol content of AMJ 0.3% v/v), or various isolated pure products (100 µM) for 24 h. (A), (B) and (C) cell apoptosis rates were assessed by flow cytometry using the annexin V-FITC/PI apoptosis assay. (D) The expression level of key regulators of cell cycle and apoptosis was assessed by Western blot analysis. The data are representative of at least three independent experiments. Values are shown as means ± S.E.M. (n = 3); *, *P<0.05* versus respective control.

Next, experiments were performed to determine whether AMJ induces apoptosis in Jurkat cells. As indicated in Fig. 2A, AMJ increased concentration-dependently both early (annexin V positive and PI negative cells) and late (annexin V and PI positive cells) apoptosis rates with a statistically significant effect observed at concentrations of or greater than 0.2% v/v.

AMJ is known to contain a mixture of polyphenolic compounds and, in particular anthocyanins and flavonols [7]. In order to identify active phenolic compounds, AMJ was subjected to fractionation using semi-preparative HPLC, yielding 10 fractions and the ability of these fractions to induce apoptosis in Jurkat cells was evaluated. Surprisingly, all fractions induced apoptosis in Jurkat cells when tested at 21.4 µg/mL, which corresponds to the polyphenol concentration in 0.3% (v/v) of AMJ (Figure 2B). The analysis of the different fractions by analytical HPLC and LC-MS techniques has lead to the tentative identification of several chlorogenic acids, cyanidin glycosides and derivatives of quercetin (Table 1). Moreover, the ability of different authentic polyphenols present in AMJ to induce apoptosis in Jurkat cells was determined. These findings indicate that cyanidin, cyanidin-3-*O*-rutinoside, cyanidin-3-*O*-arabinoside, chlorogenic acid and neochlorogenic acid significantly increased apoptosis in Jurkat cells whereas cyanidin-3-*O*-glucoside and cyanidin-3-*O*-galactoside were without effect when tested at 100 µM (Figure 2C). These findings indicate that the AMJ-induced apoptosis in Jurkat cells involves different polyphenolic molecules.

**Table 1:** Tentative identification of phenolic compounds present in AMJ-derived fractions by HPLC and LC-MS analysis.

Fraction	*t*_r_ (min)	λmax (nm)	[M]^+^ (*m/z*)	MS^2^ ions (*m/z*)	Tentative identification	Percentage area (TIC)
**1**	27	325	355	163	Neochlorogenic acid	*69%*
	45	525	419	391-279	Cyanidin-3-arabinoside	*9%*
	47	525	419	391,307,289	Cyanidin-3-xyloside	*22%*
**2**	27	325	355	163	Neochlorogenic acid	*100%*
**3**	43	525	449	391,368	Cyanidin-3-galactoside	*72%*
	45	525	419	391,338	Cyanidin-3-arabinoside	*20%*
**4**	38	325	355	163	Chlorogenic acid	*4%*
	45	525	419	305,391	Cyanidin-3-arabinoside	*92%*
	47	525	419	391	Cyanidin-3-xyloside	*4%*
**5**	38	325	355	163	Chlorogenic acid	*68%*
	45	525	449	355,391	Cyanidin-3-glucoside	*12%*
	47	525	419	391	Cyanidin-3-xyloside	*20%*
**6**	45	525	419	387,287,207	Cyanidin-3-arabinoside	*84%*
	47	525	419	391,279,201	Cyanidin-3-xyloside	*16%*
**7**	45	525	595	517,391	Cyanidin-rutinoside	*n.p.*
	45	525	419	391,309	Cyanidin-3-arabinoside	*n.p.*
**8**	43	355	517	487,303	Quercetin-hexoside	*46%*
	48	355	627	487,303	Quercetin-diglycoside	*38%*
**9**	48	355	627	465,303	Quercetin-diglycoside	*75%*
**10**	55	355	611	597,465,303	Quercetin-rutinoside	*90%*

All retention time (*t*_r_) and λmax were obtained with analytical HPLC. Mass spectra were obtained with LC-MS and the percentage of compounds in each fraction is given as percentage of AUC for Total Ion Current (TIC) trace. n.p., not performed due to co-elution of compounds in LC-MS analysis.

To characterize the AMJ-induced pro-apoptotic signaling pathway involved in G_2_/M phase cell cycle arrest, the expression level of the tumor suppressor protein p73, which is known to function as a p53 homolog in the p53-deficient Jurkat cells was determined [18], [19]. As shown in Fig. 2D, p73 was undetectable in control cells, whereas after AMJ treatment there was a concentration-dependent increase in the expression level of p73.

Cyclin B1 plays a critical role in G_2_/M cell cycle transition [20]. Therefore, the expression level of cyclin B1 in AMJ-treated Jurkat cells was assessed. AMJ caused a concentration-dependent down-regulation of the expression of cyclin B1 (Fig. 2D), which most likely contributes to the G_2_/M cell cycle arrest.

Interestingly, the up-regulation of p73 and down-regulation of cyclin B1 are associated with a marked increase in the expression of active caspase 3 (cleaved-caspase 3), one of the main executors of apoptosis (Fig. 2D). UHRF1 (Ubiquitin-like, containing PHD and RING Finger domains, 1), over-expressed in various cancers, is an essential protein required for the maintenance of DNA methylation patterns. UHRF1 regulates the G1/S transition of cell cycle and is involved in the negative regulation of certain tumor suppressor genes such as *p16^INK4A^*, and *pRB* [18], [21], [22], [23]. Treatment of Jurkat cells with AMJ caused a marked down-regulation of UHRF1 (Fig. 2D), providing further evidence for the anti-proliferative and pro-apoptotic properties of AMJ. Altogether, these findings indicate that AMJ inhibits the proliferation of Jurkat cells by inducing apoptosis likely through the p73-associated activation of caspase 3, and the down-regulation of cyclin B1 and UHRF1.

### AMJ stimulates the intracellular formation of ROS in Jurkat cells

**Figure 3:**
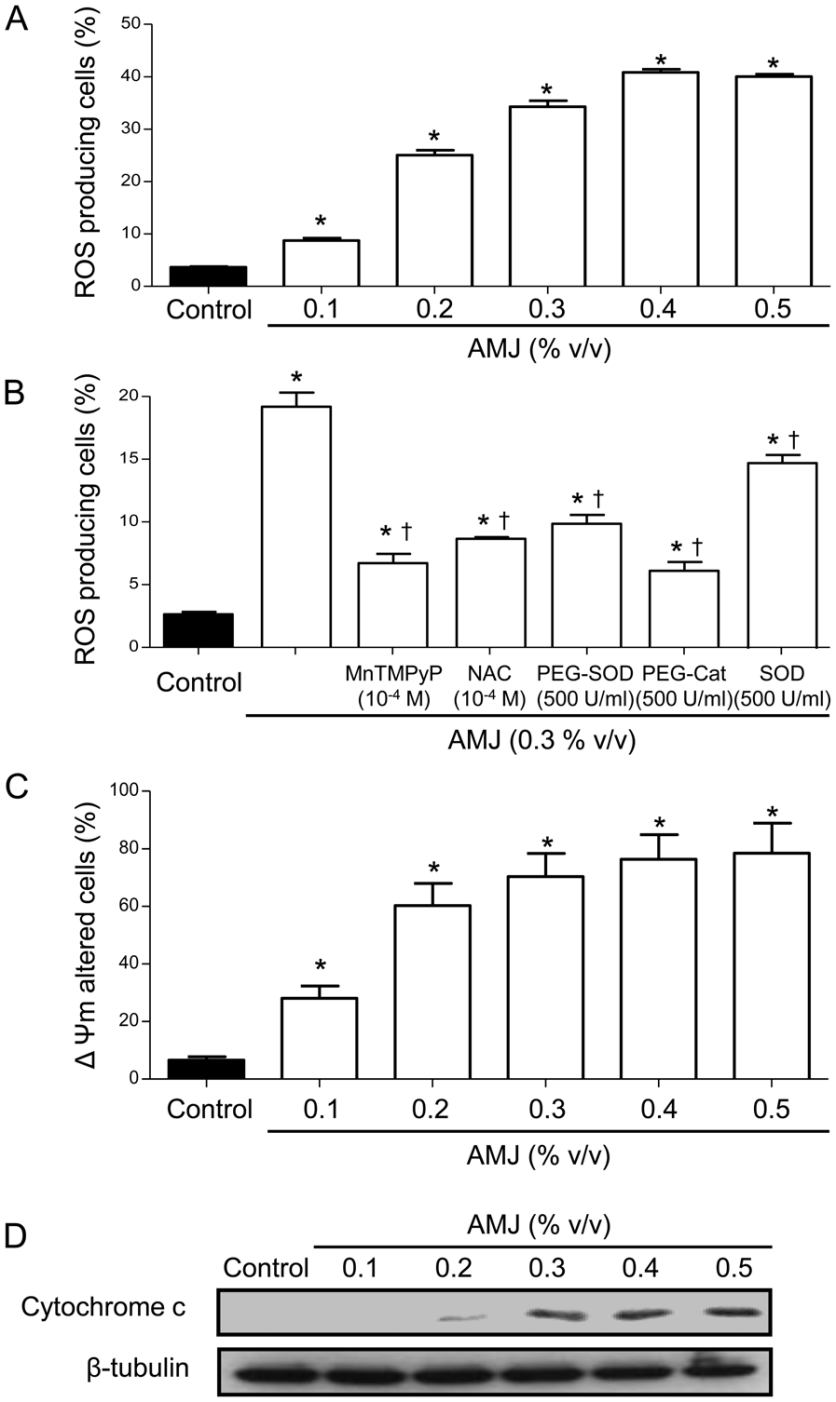
AMJ induced a pro-oxidant response and altered the ΔΨm in Jurkat cells. Cells were exposed to increasing concentrations of AMJ for 24 h. (A) and (B) The formation of ROS was assessed by flow cytometry after incubation with the redox-sensitive fluorescent probe DHE. Cells were exposed to antioxidant treatment as indicated 30 min before the addition of AMJ. (C) Changes in the ΔΨm were assessed by flow cytometry using the fluorescent probe DiOC6. Values are shown as means ± S.E.M. (n = 3); *, *P<0.05* versus control; †, *P<0.05* versus AMJ treatment. (D) The level of cytochrome c in the cytosolic fractions of Jurkat cells was assessed by Western blot analysis. Similar findings were observed in three independent experiments.

Increased steady-state levels of ROS are known indicators of the pro-apoptotic effects of various natural phytochemicals, especially polyphenols [3], [18], [24], [25], [26]. Therefore experiments were performed to determine whether the polyphenolic-rich AMJ stimulates the formation of ROS in Jurkat cells. As indicated in Fig. 3A, exposure of Jurkat cells to AMJ concentration-dependently increased the formation of ROS. To determine whether the AMJ-induced formation of ROS is an intracellular or an extracellular event, the effect of various antioxidants was examined. A marked reduction of ROS formation was observed when AMJ-treated cells were concomitantly exposed to the antioxidant compound NAC or the membrane-permeant analogs of either SOD (MnTMPyP, PEG-SOD) or catalase (PEG-catalase). In contrast native SOD, which is unable to cross membranes, only slightly but significantly, reduced the AMJ-induced production of ROS (Fig. 3B). Thus, these data indicate that AMJ predominantly stimulates the intracellular formation of ROS involving superoxide anions and hydrogen peroxide.

### AMJ induces the breakdown of ΔΨm and stimulates the mitochondrial release of cytochrome c in Jurkat cells

Loss of mitochondrial membrane potential ΔΨm is considered as one of the hallmarks of ROS-induced apoptosis [18], [26]. DiOC6, a mitochondrial specific and voltage-dependent fluorescent dye, was therefore used to determine whether AMJ affects the mitochondrial membrane potential in Jurkat cells. As shown in Fig. 3C, the number of cells emitting low fluorescence levels was significantly increased in a concentration-dependent manner by AMJ, indicating a dramatic drop of ΔΨm.

Since the mitochondrial membrane disruption is often associated with the release of mitochondrial proteins into the cytoplasm [24], the sub-cellular distribution of cytochrome c after AMJ exposure was determined. As indicated in Fig. 3D, AMJ increased in a concentration-dependent manner the level of cytochrome c detected in the cytosolic fraction of cells. Altogether, these findings indicate that AMJ induces apoptosis by generating a pro-oxidant signal and triggering mitochondrial membrane potential loss with a subsequent release of cytochrome c from mitochondria into the cytoplasm.

### The AMJ-induced apoptosis and upregulation of p73 and active caspase 3 and down-regulation of UHRF1 are redox-sensitive events in Jurkat cells

**Figure 4:**
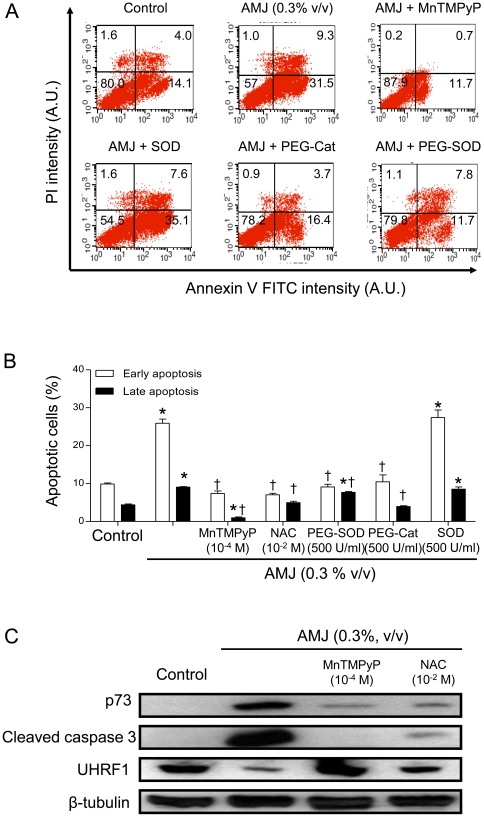
AMJ induced a ROS-dependent pro-apoptotic effect in Jurkat cells. Cells were exposed to antioxidant treatment as indicated 30 min before the addition of AMJ for 24 h. (A) and (B) cell apoptosis rates were assessed by flow cytometry using the annexin V-FITC/PI apoptosis assay. (A) Illustrates representative cell sorting results for the indicated treatments. (B) shows cumulative data of cells in early or late stages of apoptosis. Values are shown as means ± S.E.M. (n = 3); *, *P<0.05* versus respective control; †, *P<0.05* versus respective AMJ-treated cells. (C) The expression level of p73, active caspase 3 and UHRF1 was assessed by Western blot analysis. Similar findings were observed in three independent experiments.

To determine whether the intracellular formation of ROS is a major initiator of the p73-related pro-apoptotic signaling pathway activated by AMJ, the formation of ROS was inhibited by adding various antioxidants. Exposure of cells to an intracellular antioxidant (MnTMPyP, PEG-SOD and PEG-Catalase) or NAC prevented the AMJ-induced early and late apoptosis, whereas native SOD did not have such an effect (Fig. 4A and B).

Next, immunoblot analysis was performed to determine whether the redox-sensitive pro-apoptotic activity of AMJ on Jurkat cells is associated with changes in the expression level of p73, active caspase 3 and UHRF1. As shown in Fig. 4C, treatment of cells with MnTMPyP or NAC abolished the AMJ-induced up-regulation of p73, active caspase 3, and down-regulation of UHRF1.

In order to obtain evidence whether or not changes in UHRF1 are a key event for the AMJ-induced induction of apoptosis in Jurkat cells, we have performed additional experiments by over-expressing UHRF1. The data indicate that over-expression of UHRF1 using pSG5-UHRF1 plasmid did not reduce and/or abolish the AMJ-induced apoptosis (data not shown). Moreover, our recent findings also indicate that caspase 3 is involved in the degradation of UHRF1 [18]. Thus, these data suggest that UHRF1 is only one of the down-stream effectors of the redox-sensitive up-regulation of p73 and activation of caspase 3 in the AMJ-induced apoptotic pathway. Altogether, these findings indicate that AMJ triggers apoptosis in Jurkat cells through redox-sensitive activation of a p73-related caspase 3-dependent pro-apoptotic pathway.

### AMJ induces apoptosis in human lymphoblastic leukemia cells, but not in normal lymphocytes

**Figure 5:**
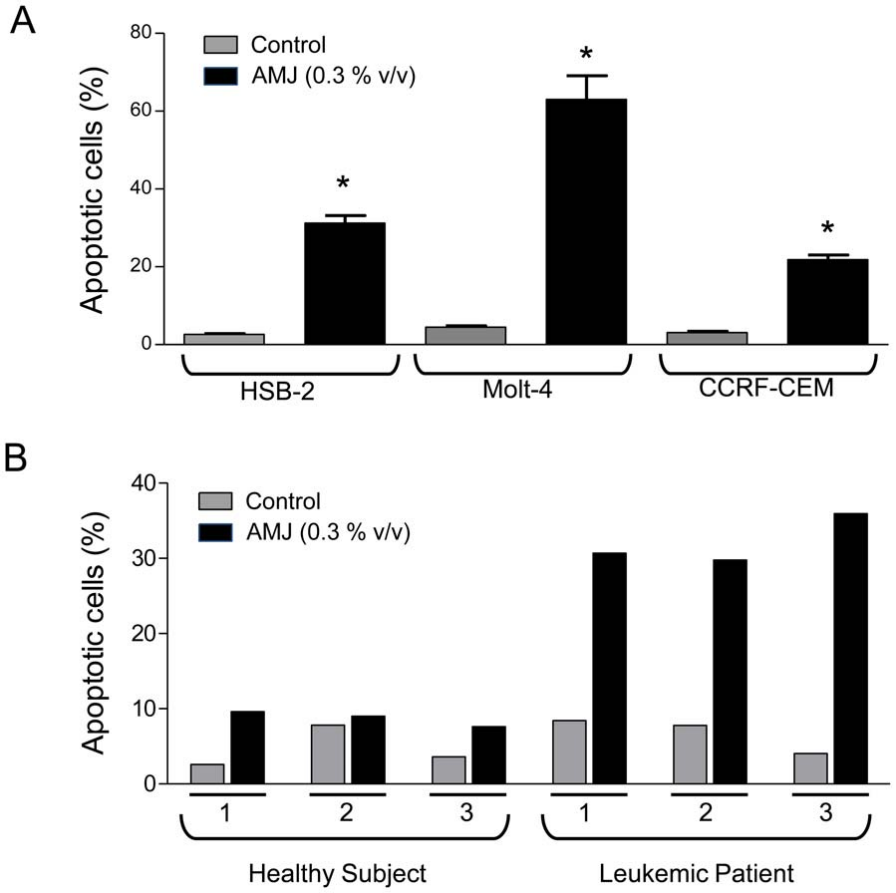
AMJ induced selective pro-apoptotic effects in different human leukemia cells and human primary lymphoblastic leukemia cells. (A) Pro-apoptotic effect of AMJ was assessed on various human leukemia cells (HSB-2, Molt-4 and CCRF-CEM). Values are shown as means ± S.E.M. (n = 3); *, *P<0.05* versus respective control; (B) The apoptosis rate of untreated or AMJ-treated primary T-lymphocytes from 3 healthy subjects or lymphoblastic leukemia cells from 3 patients was assessed by flow cytometry using the annexin V-FITC/PI apoptosis assay. The bar graph shows the cumulative percentage of cells which are in apoptosis.

To determine whether AMJ induces pro-apoptotic effects also in other types of human lymphoblastic leukemia cells, besides Jurkat cells, we performed experiments with HSB-2, Molt-4 and CCRF-CEM cell lines. As shown in Fig. 5A, AMJ at a concentration of 0.3% v/v significantly increased apoptosis in the three different leukemia cell lines. These results indicate that AMJ is able to promote apoptosis in different types of human leukemic cells. Furthermore, in order to determine the clinical relevance of the present findings, the pro-apoptotic effect of AMJ was determined on human primary acute lymphoblastic leukemia cells as well as normal human primary T-lymphocytes. As shown in Fig. 5B, AMJ at a concentration of 0.3% v/v induced apoptosis in lymphoblastic leukemia cells by about 30% whereas little effect was observed in normal lymphocytes. These findings indicate that AMJ preferentially induces apoptosis in lymphoblastic cells, which exhibit a cancer signature.

## Discussion

Polyphenols from natural sources present in plant materials have been suggested to have cancer chemopreventive and chemotherapeutic properties [1], [2], [27], [28]. In particular, epidemiological studies have highlighted the important role of polyphenol-containing fruit and vegetable consumption in protecting individuals from developing certain cancers including gastrointestinal cancer and leukemia [16], [17], [29]. Besides fruits and vegetables, berries, in particular black and red berries, are particularly rich in polyphenols, which may have a beneficial anticancer effect [1]. Indeed, extracts from the black chokeberry, *Aronia melanocarpa* [8] have been shown to have pronounced anticancer effects both in *in vivo* and *in vitro* studies on various colorectal cancer models [9], [10], [11], and leaves extracts of *Aronia melanocarpa* have been shown active on a non-solid tumor cell line, the p53-null promyeloblastic leukemia HL-60 cell line [30]. An extract of mulberry fruit also induced human glioma cell death and reduced glioma tumor growth *in vivo* [31]. Moreover, black raspberry and blueberry reduced estrogen-induced mammary tumorigenesis [32], and bilberry, lingonberry and cloudberry reduced adenoma formation in Apc- mutated Min/+ mice [33]. The major aim of the present study was to determine the anticancer effect of *Aronia melanocarpa* fruit juice, a rich source of polyphenols (7.15 g/L), on the acute lymphoblastic leukemia Jurkat cell line, which is deficient for p53 [34], and, if so, to characterize the mechanism involved and to identify active polyphenols involved in the AMJ-induced effects.

The present study indicates that *Aronia melanocarpa* juice markedly inhibited the proliferation of leukemic Jurkat cells in a concentration-dependent manner. The growth inhibitory effect is associated with an arrest of the cell cycle progression in G_2_/M phase as shown by the cell phase distribution, and a marked down-regulation of the G_2_/mitotic-specific cyclin B1 [20]. In addition, AMJ promoted in a concentration-dependent manner these cells towards apoptosis as indicated by annexin V labeling. In contrast, the red wine polyphenol-induced inhibition of proliferation and induction of apoptosis in Jurkat cells is associated with a cell cycle arrest in G0/G1 [3]. This difference in cell cycle distribution between AMJ and red wine polyphenols may be due to the different phenolic composition between these two sources.

In order to characterize the mechanism involved in the pro-apoptotic signaling pathway activated by AMJ, the expression status of the cell cycle checkpoint regulator p73 was determined. Indeed, the p53-homolog p73 has been shown to act as a salvage cellular gatekeeper in p53-deficient cells such as the Jurkat cells [18], [19], [35]. The present findings indicate that AMJ caused a marked and concentration-dependent up-regulation of p73, suggesting that this cell cycle checkpoint regulator is a key mediator of the pro-apoptotic activity of AMJ in Jurkat cells. In addition, AMJ also caused a concomitant down-regulation of the epigenetic integrator UHRF1. Similarly, red wine polyphenols have been shown to induce the p73-dependent pro-apoptotic pathway and the dowregulation of UHRF1 in Jurkat cells [3]. Moreover, a recently published study has shown that depleting cancer cells of UHRF1 causes cell cycle arrest in G2/M and apoptosis [36]. UHRF1 is a member of a subfamily of RING-finger type E3 ubiquitin ligases known to bind to methylated DNA [37] and to recruit the DNA methyltransferase-1 [38] to regulate cell cycle progression [39] and gene expression [40]. The present findings indicate that AMJ triggers G2/M cell cycle arrest and apoptosis of the p53-deficient lymphoblastic leukemia Jurkat cell through a p73-associated pathway leading to the down-regulation of UHRF1. The pro-apoptotic activity of AMJ is associated with the concentration-dependent expression of active caspase 3, a major effector of the p73-related pro-apoptotic pathway [18]. In our previous study, we have shown that UHRF1 is a down-stream target of caspase 3 in Jurkat cells [38]. Altogether, these findings indicate that AMJ is able to induce apoptosis in leukemic cells through a caspase 3-dependent pro-apoptotic pathway despite the absence of the powerful endogenous anticancer gatekeeper p53. They further suggest that AMJ is likely to have chemotherapeutic activity against leukemias, including those (about 60%) exhibiting resistance to p53-dependent apoptosis due to mutations in the p53 tumor suppressor gene [41]. In order to obtain evidence for the clinical relevance, the pro-apoptotic effect of AMJ was studied in human primary lymphoblastic leukemia and normal human primary T-lymphocytes cells. These findings indicate that AMJ selectively killed leukemic cells but affected minimally normal T-lymphocytes. The reason for the selective pro-apoptotic activity of AMJ towards cancer cells remains unclear. One possible explanation, which is in agreement with the Warburg's concept, may be related to the fact that transformed cells have a higher metabolic rate than normal cells [42], [43]. The Warburg effect is considered as one of the most fundamental alteration during malignant transformation, and expects that any cancer cell displays enhanced metabolic activity due to elevated levels of glycolysis [42], [43].

One of the major findings of the present study is that AMJ exerted its anticancer activity on Jurkat cells by causing a sustained pro-oxidant response as assessed using a redox-sensitive fluorescent probe. The pro-oxidant response to AMJ was prevented by membrane-permeable mimetics of either superoxide dismutase or catalase, but little or not at all by native superoxide dismutase, indicating the involvement of both intracellular superoxide anions and hydrogen peroxide. The pro-oxidant response appears to be a key event in the AMJ-induced apoptosis in Jurkat cells since intracellular scavengers of ROS markedly reduced AMJ-induced apoptosis and the up-regulation of p73, active caspase 3 expression and the subsequent down-regulation of UHRF1. Thus, AMJ is acting as a powerful intracellular inducer of ROS formation, which exerts its anticancer effects by inhibiting cell growth, arresting cell cycle progression in G2/M and inducing a p73-related caspase 3-dependent apoptosis in Jurkat cells. A similar pro-oxidant mechanism has also been involved in the red wine polyphenol-induced cell cycle arrest in G0/G1 and induction of apoptosis in Jurkat cells [3]. In addition, previous studies have indicated that resveratrol [27], epigallocatechin-3-gallate [2], [44] and curcumin [28] inhibited the proliferation of various types of cancer cells through a pro-oxidant response involving the intracellular formation of ROS. These ROS might be predominantly originating from the mitochondrial respiration chain and also from reduced oxidative defense mechanisms subsequent to the down-regulation of mitochondrial superoxide dismutase [45], [46]. Therefore, experiments were performed to test whether the AMJ-induced pro-oxidant response involves the mitochondria. Such a possibility is supported by the fact that AMJ induced a concentration-dependent ΔΨm disruption and the release of cytochrome c from mitochondria into the cytoplasm in Jurkat cells.

In addition, the fractionation of AMJ by preparative HPLC and the use of commercially available isolated compounds have indicated that the pro-apoptotic effect of AMJ involves different polyphenolic compounds such as chlorogenic acids from the hydroxycinnamic acid sub-class, cyanidin glycosides from the anthocyanin sub-class and derivatives of quercetin from the flavonol sub-class. The involvement of several active phenolic compounds in the pro-apoptotic effect of AMJ is consistent with previous studies indicating anti-cancer properties of a wide range of phenolic structures such as anthocyanins [10], curcumin [28], resveratrol [27] and catechins [44].

In conclusion, the present findings indicate that the polyphenol-rich AMJ effectively and selectively induced programmed cell death of T cell-derived lymphoblastic leukemia cells. The anticancer activity is critically dependent on the intracellular formation of ROS involving superoxide anions and hydrogen peroxide, which, in turn, regulate the expression of key regulators of G_2_/M cell cycle transition and apoptosis.

## Materials and Methods

### Ethics Statement

Primary human lymphoblastic leukemia cells were obtained from patients who gave their written consent according to the principles expressed in the Declaration of Helsinki. The study has been approved by the local ethics committee for biomedical research, the “Comité de Protection des Personnes” (CPP EST-IV).

### Preparation of AMJ

Aronia juice concentrate (66° Bx) was provided by Eckes-Granini (Nieder-Olm, Germany). AMJ was reconstituted by dilution to 15° Bx in distilled water. The final aronia juice contained 7.15 g/L polyphenols expressed as gallic acid equivalents measured by the Folin-Ciocalteu method [47].

### Cell culture, treatments and transfection

The Jurkat, HSB62, Molt-4 and CCRF-CEM cell lines were obtained from the American Type Culture Collection (Manassas, VA, USA) and maintained in a humidified incubator with 5% CO_2_ at 37°C, as previously described [40]. Cells were cultured in RPMI 1640 supplemented with 10% (v/v) fetal bovine serum (Lonza Biowhittaker, Verviers, Belgium), 2 mM L-glutamine, 100 U/mL penicillin and 100 µg/mL streptomycin (Sigma-Aldrich, St Louis, MO, USA). Primary human T-lymphocytes were separated from blood of healthy donors by using the Pan T Cell Isolation Kit II –human- (Miltenyi Biotec SAS, Paris, France) and were maintained in supplemented RPMI 1640. Primary human lymphoblastic leukemia cells obtained from patients, which gave their written consent, were separated from blood using the Lymphocyte Separation Medium LSM 1077 (PAA Laboratories, Les Mureaux, France) and maintained in supplemented RPMI 1640. Cells were grown for 24 h and then they were exposed to AMJ at different concentrations for an additional 24 h. In some experiments, different antioxidants (manganeseIII tetrakis1-methyl-4-pyridyl porphyrin or MnTMPyP, Enzo Life Sciences; N-acetyl cysteine or NAC; superoxide dismutase or SOD; polyethylene glycol-superoxide dismutase or PEG-SOD; polyethylene glycol-catalase or PEG-catalase, Sigma-Aldrich) were added to the culture medium 30 min before addition of AMJ.

### MTS assay

Jurkat cells (2×10^4^) were seeded in 96-well microplates, grown for 24 h and then exposed to AMJ at different concentrations in triplicate. After 24 h, 20 µL of MTS reagent (CellTiter 96® Aqueous One Solution, Promega, Charbonnières-Les-Bains, France) was added to each well for 2 h. Absorbance was then measured at 490 nm using a multiwell ELISA plate reader. The percentage of living cells was calculated as a ratio of the OD value of each AMJ-treated cell sample to the OD value of the corresponding control.

### Cell cycle phase distribution analysis

Jurkat cells were seeded in 80 cm^2^ culture flasks at a density of 2×10^5^ cells/mL, grown for 24 h and then exposed to AMJ at different concentrations for 24 h. Thereafter, cells were collected, washed and fixed in 70% ethanol. After incubation for at least 2 h at 4°C, cells were washed, treated with a RNase solution (Sigma-Aldrich) and stained with the DNA fluorochrome propidium iodide (PI, 50 µg/mL, Sigma-Aldrich) for 30 min at room temperature. Propidium iodide fluorescence was then measured by flow cytometry (FACScan, BD Biosciences, San Jose, CA, USA). A minimum of 20,000 cells were acquired per sample, and the data were analyzed using the Modfit software. The percentage of cells in G_1_, S and G_2_/M was determined from DNA content histograms.

### Apoptosis analysis

The Annexin V-FITC/PI Apoptosis Assay (BD Biosciences Pharmingen, San Diego, CA, USA) was used to detect early and late apoptosis. Annexin V has a strong affinity for phosphatidylserine, which is externalized in membranes of apoptotic cells. Experiments were performed according to the manufacturer's indications on different leukemic cell lines, primary normal T-lymphocytes or primary human lymphoblastic leukemia cells, exposed to AMJ at different concentrations for 24 h. Apoptosis rates were then assessed by flow cytometry. At least 10,000 events were recorded and represented as dot plots.

### Determination of ROS formation

The level of cellular ROS formation was determined as previously described [18]. Briefly cells, exposed for 24 h to AMJ at different concentrations, were stained with dihydroethidine (DHE) which is a redox-sensitive fluorescent probe rapidly oxidized to ethidium (a red fluorescent compound) by superoxide anions. Ethidium is then trapped in the nucleus by intercalating into DNA, leading to an increase of ethidium fluorescence intensity. After staining with DHE, cells were subjected to flow cytometric examination (BD FACSCalibur, Becton Dickinson, Franklin Lakes, NJ, USA). Data were acquired and analysed using the CellQuest software (BD Biosciences, Becton Dickinson). Histograms of 10,000 events were recorded per experiment.

### Measurement of mitochondrial membrane potential ΔΨm

Cells seeded at a density of 2×10^5^ cells/well in 6-well plates, were treated with AMJ for 24 h at increasing concentrations. The ΔΨm changes were then determined by incubating cells with 40 nM of DiOC6 (3,3′-Dihexyloxacarbocyanine iodide) and 1 µg/mL PI for 15 min prior to flow cytometry examination (FACScan, BD Biosciences). For the DiOC6 stained samples, PI-positive cells were excluded. At least 5,000 cells were analyzed for each sample.

### Western blot analysis

Proteins from whole cell lysates or from cytosolic fractions of AMJ treated or untreated cells were extracted, separated on 10% SDS-polyacrylamide gels and transferred onto PVDF membranes as previously described [18], [48]. Membranes were then probed overnight at 4°C with the appropriate primary antibodies (mouse monoclonal anti-p73 antibody, BD Biosciences Pharmingen; rabbit polyclonal anti-cleaved caspase 3 antibody, Cell Signaling Technology, Danvers, MA, USA; mouse monoclonal anti-cyclin B1 antibody -BD Biosciences Pharmingen-; mouse monoclonal anti-UHRF1 antibody [18]; mouse monoclonal anti-cytochrome c, BD Biosciences Pharmingen). Membranes were thereafter incubated for 1 h with the corresponding horseradish peroxidase linked secondary antibody. Immunoreactive bands were detected using ECL chemiluminescence substrate solution (GE Healthcare Europe GmbH, Saclay, France). Membranes were stripped subsequently and reprobed with a mouse monoclonal anti-beta tubulin antibody (BD Biosciences Pharmingen).

### Preparative HPLC

AMJ was fractionated using preparative HPLC. Separations were carried out using a semipreparative RP-HPLC system comprising a Gilson 305 pump and a Gilson 115 UV detector (Gilson International-France, Roissy, France) fitted with a column C18-Nucleodur®, 250×21 mm; 10 µm, (Macherey-Nagel, Hoerdt, France), and eluted with 0.01% aqueous formic acid (A) and methanol (B) at a flow rate of 14 mL/min following the conditions: from 90% to 50% A for 80 min, and 100% of B for 5 min, followed by washing and reconditioning of the column. Fractions were monitored at 370 nm and each individual large peak (Fractions 1 to 10) was collected separately. All fractions were reduced to dryness using a rotary evaporator set at 40°C.

### Analytical RP-HPLC

All fractions were analyzed using an analytical RP-HPLC system comprising a Varian 9010 pump and a Varian ProStar® 330 diode array (Varian, Courtaboeuf, France) fitted with a column C18-Nucleodur®, 250×4.6 mm; 5 µm (Macherey-Nagel, Hoerdt, France) and eluted with water containing 0.1% TFA (A) and methanol (B) using the following gradient: from 90% to 50% A for 50 min, 50% A for 5 min and 100% B for 10 min and back to the initial conditions. The flow rate was 1 mL/min at 25°C.

### LC-MS analysis

Identification of major phenolic compounds in fractions was carried out by LC-MS technique on an Agilent 1200 SL HPLC (Agilent, Massy, France) chain fitted with a 30×1 mm; 1.9 µm i.d. Hypergold column (Thermo Scientific, Courtaboeuf, France) and coupled to a HCT ultra mass spectrometer (Bruker Daltonics, Wissembourg, France) and eluted with acetonitrile (A) and water (B) using the following gradient: from 98% to 2% A in 6 min, followed by washing and reconditioning of the column. All analyses were done in positive ionisation using data dependent MS^2^. The tentative identification of compounds present in the fractions was based both on the comparison of absorbance spectra and mass spectra, using MS^2^, with those of *Aronia melanocarpa* phenolic compounds previously reported in the literature [7], and on a co-chromatography with authentic standards, whenever available. Quantitative estimations of proportions are based on the area under the curve for total ion signal on MS analysis.

### Isolated pure products

Pro-apoptotic effect of isolated pure products present in the various fractions of AMJ was also studied. Cyanidin, cyanidin-3-*O*-glucoside, caynidin-3-*O*-galactoside and cyanidin-3-*O*-rutinoside were purchased from Extrasynthese (Genay, France), whereas cyanidin-3-*O*-arabinoside was obtained from Polyphenols Ltd. (Norway) and chlorogenic acid and neochlorogenic acid were from Sigma.

### Statistical analysis

All values are expressed as means ± S.E.M. of three independent experiments. Statistical evaluation was performed with one-way ANOVA test followed by a Tukey's post-hoc test as appropriate using Graphpad Prism software (version 5.03 for Windows, Graphpad Software Inc., La Jolla, CA, USA). *P*<0.05 was considered as significant.
